# Longitudinal Structural MRI in Neurologically Healthy Adults

**DOI:** 10.1002/jmri.27203

**Published:** 2020-05-29

**Authors:** Sarah Gregory, Keith R. Lohse, Eileanoir B. Johnson, Blair R. Leavitt, Alexandra Durr, Raymund A.C. Roos, Geraint Rees, Sarah J. Tabrizi, Rachael I. Scahill, Michael Orth

**Affiliations:** ^1^ Huntington's Disease Research Centre, Institute of Neurology University College London London UK; ^2^ Wellcome Centre for Human Neuroimaging, Institute of Neurology University College London London UK; ^3^ Department of Health, Kinesiology, and Recreation University of Utah Salt Lake City Utah USA; ^4^ Department of Physical Therapy and Athletic Training University of Utah Salt Lake City Utah USA; ^5^ Centre for Molecular Medicine and Therapeutics, Department of Medical Genetics University of British Columbia Vancouver British Columbia Canada; ^6^ APHP Department of Genetics, Pitié‐Salpêtrière University Hospital, and Institut du Cerveau et de la Moell épinière (ICM) Sorbonne Université Paris France; ^7^ Department of Neurology Leiden University Medical Centre Leiden The Netherlands; ^8^ Institute of Cognitive Neuroscience University College London London UK; ^9^ Department of Neurology Ulm University Hospital Ulm Germany; ^10^ Neurozentrum Siloah Bern Switzerland

**Keywords:** diffusion, cortical thickness, cortical volume, reliability, statistical power

## Abstract

**Background:**

Structural brain MRI measures are frequently examined in both healthy and clinical groups, so an understanding of how these measures vary over time is desirable.

**Purpose:**

To test the stability of structural brain MRI measures over time.

**Population:**

In all, 112 healthy volunteers across four sites.

**Study Type:**

Retrospective analysis of prospectively acquired data.

**Field Strength/Sequence:**

3 T, magnetization prepared – rapid gradient echo, and single‐shell diffusion sequence.

**Assessment:**

Diffusion, cortical thickness, and volume data from the sensorimotor network were assessed for stability over time across 3 years. Two sites used a Siemens MRI scanner, two sites a Philips scanner.

**Statistical Tests:**

The stability of structural measures across timepoints was assessed using intraclass correlation coefficients (ICC) for absolute agreement, cutoff ≥0.80, indicating high reliability. Mixed‐factorial analysis of variance (ANOVA) was used to examine between‐site and between‐scanner type differences in individuals over time.

**Results:**

All cortical thickness and gray matter volume measures in the sensorimotor network, plus all diffusivity measures (fractional anisotropy plus mean, axial and radial diffusivities) for primary and premotor cortices, primary somatosensory thalamic connections, and the cortico‐spinal tract met ICC. The majority of measures differed significantly between scanners, with a trend for sites using Siemens scanners to produce larger values for connectivity, cortical thickness, and volume measures than sites using Philips scanners.

**Data Conclusion:**

Levels of reliability over time for all tested structural MRI measures were generally high, indicating that any differences between measurements over time likely reflect underlying biological differences rather than inherent methodological variability.

**Level of Evidence:**

4.

**Technical Efficacy Stage:**

1.

BRAIN MORPHOLOGY and anatomical connectivity are central to the in vivo characterization of biological mechanisms underlying neurodegeneration. Reliably measuring both structure and connectivity can provide information regarding the nature of disease‐related changes that occur across the course of disease progression. This can improve our understanding of the way in which pathology impacts not only structure, but also brain activity and behavior, while such changes can also act as valuable markers for both the timing and efficacy of therapeutic treatment at an exploratory level.

In the field of neuroimaging, higher‐resolution structural magnetic resonance imaging (MRI) is used to examine brain macrostructure including volume, cortical thickness, and surface area, while diffusion‐weighted MRI is one method that interrogates the microstructural properties of white matter.^1^ Both techniques are employed to measure either region‐specific or whole‐brain structural changes across clinical groups, including those with neurodegenerative disease.^2^, ^3^ In particular, structural MRI can be used to highlight and index morphological differences in regions of the brain associated with specific pathologies, as compared to healthy controls (or other disease‐groups), and how these differences change over the course of a disease trajectory.^4^ There is very robust structural MRI evidence, for example, of striatal degeneration in the early stages of Huntington's disease (HD)^5^, ^6^ and in the medial temporal lobe in probable Alzheimer's disease (AD),^7^, ^8^ while structural measures can inform clinical diagnosis and treatment decisions in disorders such as multiple sclerosis and dementia.^9^, ^10^ Structural MRI‐derived measures of brain volume are currently included as secondary endpoints in clinical trials. However, while routinely used in research studies, due to the complexity of diffusion‐weighted MRI acquisition and analysis, microstructural measures have not yet been used in clinical trials.

Despite the clear utility of both structural and diffusion‐weighted MRI, it is also important to consider that, as with any imaging technique, both noise and variations can be introduced at almost any stage. Rigorous protocol standardization and staff training notwithstanding, potential sources of variation include local magnetic field inhomogeneities, experimenter bias, inherent issues within analysis packages, and, importantly, differences between the individuals being scanned. All of these causes of variability are unrelated to the hypotheses that are being tested, but need to be considered when interpreting data. Multisite studies are becoming more frequent and they customarily include several different scanner types and models, in addition to variation in site personnel. Hence, a sound understanding of the nature and subsequent prevention of intersite differences is increasingly important.

Promisingly, there is some evidence to suggest that both standard scalar diffusion tensor imaging (DTI) metrics including diffusivity and fractional anisotropy (a ratio of diffusivity perpendicular and parallel to the direction of the main underlying fiber) and volumetric measures are reproducible across sites and scanner types despite predictably greater between‐site variability.^11^, ^12^, ^13^ However, most investigations have tended to use imaging phantoms to assess MRI reproducibility or small groups of healthy controls over very short time intervals.^11^, ^12^, ^13^ While useful, this does not provide sufficient guidance for large multisite observational studies or clinical trials that examine participants over intervals of up to a year or longer.^14^, ^15^ It is important to have a clear grasp of the reliability of structural measures in order to understand disease progression (or its modification by treatment) over substantial time intervals. This consideration is especially important in populations where brain structure is known to change over time (eg, in neurodegenerative populations). Therefore, investigating reproducibility of imaging measures in healthy volunteers over longer time periods can help characterize variability over time due to measurement error or systematic biological change (eg, “healthy” age‐related change). This, in turn, can help distinguish genuine effects related to pathology in disease populations. Accordingly, a recent study retrospectively examined the reliability of electrophysiological data in healthy individuals from the Track‐On HD multisite longitudinal study over three annual timepoints using intraclass correlation coefficients (ICCs) and demonstrated that some measures met the criteria for high levels of stability, while others did not.^16^ The study also identified limited between‐site differences.

Here we have similarly retrospectively investigated the reliability of structural and diffusion MRI‐based metrics of volume, cortical thickness, and anatomical connectivity focusing on the sensorimotor network in a similar cohort of healthy individuals from TrackOn‐HD.^2^, ^17^ We aimed to characterize the effects of time, scanner type, and site on structural brain MRI measures.

## Materials and Methods

### Participants

Healthy control participants were recruited into the TrackOn‐HD study at four study sites (London, Paris, Leiden, Vancouver).^2^, ^17^ For the present analyses, we used data from 112 participants (F = 67; mean age = 48 years ± SD: 11 years) who had complete DTI data for all three timepoints. Exclusion criteria included age below 18 or above 65 (unless previously in the Track‐HD study), major psychiatric, neurological, or medical disorder or a history of severe head injury.^17^ The study was approved by the local Ethics Committees, and all participants gave written informed consent according to the Declaration of Helsinki. At visits one to three, individuals had an average age of 48.1 years (SD: 10.7), 49.4 years (SD: 10.5), and 51 years (SD: 10.3), respectively. Attrition rates were low. At visit two, retention of participants was 93% and at visit three 87%. Table 1 contains demographic information about participants broken down by study site and scanner.

**TABLE 1:** Descriptive Information for the Sample at Each Study Site

	Leiden	London	Paris	Vancouver
Age – M (SD)	49 (10)	48 (9)	47 (12)	48 (11)
	Study site			
Total sample size – *N*	28	29	29	26
				
Sex – F (M)	19 (9)	18 (11)	15(14)	15(11)
Education – ISCED				
ISCED (1: Primary)	0	0	0	0
ISCED (2: Lower Secondary)	6	1	2	2
ISCED (3: Upper Secondary)	3	3	6	11
ISCED (4: Post Secondary)	11	11	11	5
ISCED (5: Tertiary 1)	7	13	7	10
ISCED (6: Tertiary 2)	1	1	0	0
Handedness – R	27	27	22	26
				
Scanner Make	Philips	Siemens	Siemens	Philips

### MRI Data Acquisition and Analysis

Data acquisition across sites was standardized as previously described.^2^, ^18^ In short, all site staff participated in a training session and regular contact was maintained between sites and study coordination throughout data collection. Prior to the start of the study, a human phantom was used at all sites to ensure identical settings and instructions. Throughout the study, data quality was monitored visually by IXICO (UK; Contract Research Organization). In parallel, quality control (QC) software was applied to all scans within 3 working days of acquisition. 3T MRI data were acquired on two different scanner systems (Philips Achieva at Leiden, Netherlands, and Vancouver, British Columbia, Canada, and Siemens TIM Trio at London, UK, and Paris, France). T_1_‐weighted image volumes were acquired using a 3D magnetization prepared rapid gradient echo (MPRAGE) acquisition sequence with the following imaging parameters: relaxation time (TR) =2200 msec (Siemens [S]) / 7.7 msec (Philips [P]), echo time (TE) = 2.2 msec (S) / 3.5 msec (P), flip angle (FA) = 10^o^(S)/8^o^(P), field of view (FOV) = 28 cm (S) / 24 cm (P), matrix size 256 × 256(S)/224 × 224(P), 208(S)/164(P) sagittal slices with a slice thickness of 1.0 mm with no gap. Diffusion‐weighted images were collected with 42 unique gradient directions (*b* = 1000 sec/mm^2^) on both scanner types plus eight images with no diffusion weighting (*b* = 0 sec/mm^2^) (S) and one image with no diffusion weighting (*b* = 0 second/mm^2^) (P). Acquisition parameters were TE = 88 msec, TR = 13,000 msec, and voxel size 2 × 2 × 2 mm (S); TE = 56 msec and TR = 11 sec, and voxel size 1.96 × 1.96 × 2.75 mm (P).

### T_1_ Processing

T_1_ scans underwent visual QC upon data collection (performed by S.G., E.J., R.S.) to check for incorrect parameters in the metadata and image artifacts such as motion artifacts. Scans were then bias‐corrected to correct for inhomogeneity within the images using the N3 algorithm.^19^ Images were segmented using FreeSurfer v. 5.3 run via the default recon‐all pipeline with the 3T flag. FreeSurfer has two independent default automatic processing streams surface‐ and volume‐based used to calculate different characteristics of structural MRI scans. Following processing, all FreeSurfer regions underwent visual QC (performed by S.G., E.J., R.S.), with both volumetric and thickness regions examined and scans excluded if regions showed a high degree of error across multiple slices. Volumetric and thickness values were automatically calculated and extracted from the following Brodmann areas: BA1 (somatosensory area), BA2 (somatosensory area), BA3a (somatosensory area), BA4a (primary motor area; anterior), BA4p (primary motor area; posterior), and BA6 (premotor area).

### DTI Processing

Diffusion data were preprocessed using standard FSL (FMRIB Software Library) pipelines https://fsl.fmrib.ox.ac.uk/fsl/fslwiki.^20^ Each DTI dataset was screened for artifacts (performed by S.G., E.J., R.S.), signal dropout and motion and then motion‐corrected using eddy_correct in FSL; vector gradient information was updated accordingly. Both the B_0_ and T_1_ structural images were skull‐stripped using the Brain Extraction Tool (BET) and manually corrected for instances of mis‐segmentation, whereby extraneous tissue had not on occasion been removed. To improve quality of the extracted structural image, we combined and dilated a thresholded segmented image with an eroded brain‐extracted T_1_ mask, which was then applied to the original brain‐extracted T_1_ image. The new T_1_ image was then registered to the B_0_ image using FMRIB's Linear Image Registration Tool. Diffusion tensors were fit using dtifit and crossing fibers modeled using Bedpostx.^21^ Probabilistic tractography was then performed for a series of sensorimotor tracts using probtrackx^22^; these included tracts connecting the primary motor cortex (M1) and the motor thalamus; the premotor cortex (PMC) and the motor thalamus; and the primary somatosensory cortex (S1) and the somatosensory thalamus. Regions of interest were created using the Anatomy Toolbox and warped into native space for each participant. Exclusion masks were used to exclude streamlines from outside the anatomically‐defined tract and a white matter termination mask to ensure tracts did not extend into gray matter, cerebrospinal fluid (CSF), or dura. All tracts were then warped into diffusion space using FLIRT. Fractional anisotropy (FA), mean diffusivity (MD), axial diffusivity (AD), and radial diffusivity (RD) were extracted for each participant for each tract.

### Statistical Analysis

To assess the reliability of our MRI measures over time, we calculated the *average* two‐way random‐effects intraclass correlation coefficient for absolute agreement (ICC), hereafter written as *ICC(2*,*k)*. For the two‐way random‐effects ICC, participants and observations were treated as random effects (ie, we assumed that both people and timepoints were samples from a larger population^23^). For each dependent variable, data were filtered prior to analysis to remove participants with missing data (the number of missing cases is reported for each variable below). The ICC(2,k) can be interpreted as the ratio of true variance to total variance for *k* measures.^24^ In our case, k = 3 for the three timepoints and we selected ICC(2,k) ≥0.80 as the cutpoint indicating a relatively stable and reliable measure with relatively little variation within a person over time compared to the individual differences between people.^16^, ^25^ The *single* measure two‐way random‐effects ICC was calculated to estimate absolute agreement, referred to as ICC (2,1).

To establish systematic sources of variation in our data, we also conducted 3 × 2 repeated measures analysis of variance with a within‐participant factor of time and a between‐participant factor of scanner type. For these tests, we applied a Bonferroni correction for multiple comparisons to each effect in the model (ie, the alpha‐level for main‐effects and interactions was adjusted independently). Mauchly's test was used to check for violations in sphericity. If violations were found, a Greenhouse–Geisser correction was applied (denoted by adjusted degrees of freedom in the tables below). Within each scanner type, we also conducted pairwise comparisons comparing the different study sites using the same scanner to each other. All analyses were conducted using SPSS v. 23.0 (IBM, Armonk, NY). All descriptive statistics are reported as mean (SD) unless otherwise indicated.

## Results

### Assessing Reliability over Time

All measures of white matter diffusivity (AD, RD, FA, MD) in connections between the M1 motor cortex region, the premotor cortex, or the S1 somatosensory cortex and the thalamus as well as the cortico‐spinal tract met the ICC(2,k) cutoff ≥0.80, indicating high reliability (Table 2). In addition, all measures of cortical thickness and volume of various cortical gray matter regions in both hemispheres (BA1, BA2, BA3a, BA3b, BA4a, BA4p, BA6) also met the ICC(2,k) cutoff criterion ≥0.80 of high reliability (Tables 3 and 4, respectively). It should also be noted that ICC (2,1) was moderate to high across all measures. However, a number of measures scored between 0.6–0.8.

**TABLE 2:** Reliability of Structural Connectivity Measures

Connectivity measures	Time1	Time2	Time3	Missing cases	ICC	ICC	Main effect of time
	mean (SD)	mean (SD)	mean (SD)		(2,k)	(2,1)	
CST_AD	1.18E‐03 (4.14E‐05)	1.18E‐03 (4.18E‐05)	1.18E‐03 (3.82E‐05)	48	0.95	0.87	F(2,124) = 1.18, *P* = 0.31
CST_FA	5.25E‐01 (2.33E‐02)	5.27E‐01 (2.34E‐02)	5.24E‐01 (2.30E‐02)	48	0.91	0.77	F(2,124) = 1.85, *P* = 0.16
CST_MD	7.16E‐04 (2.37E‐05)	7.12E‐04 (2.32E‐05)	7.12E‐04 (1.88E‐05)	48	0.92	0.79	F(2,124) = 2.88, *P* = 0.06
CST_RD	4.83E‐04 (2.43E‐05)	4.78E‐04 (2.37E‐05)	4.19E‐04 (2.02E‐05)	48	0.89	0.72	F(2,124) = 3.20, *P* = 0.04
M1_Thal_AD	1.12E‐03 (4.71E‐05)	1.12E‐03 (4.97E‐05)	1.13E‐03 (5.23E‐05)	43	0.97	0.92	F(2,134) = 8.97, *P* < 0.001^a^ ^,^ ^b^
M1_Thal_FA	4.75E‐01 (2.61E‐02)	4.76E‐01 (2.67E‐02)	4.76E‐01 (2.66E‐02)	43	0.92	0.79	F(2,134) = 0.52, *P* = 0.60
M1_Thal_MD	7.15E‐04 (2.46E‐05)	7.17E‐04 (2.67E‐05)	7.20E‐04 (2.50E‐05)	43	0.95	0.85	F(2,134) = 2.85, *P* = 0.06^b^
M1_Thal_RD	5.14E‐04 (2.42E‐05)	5.13E‐04 (2.60E‐05)	5.15E‐04 (2.30E‐05)	43	0.9	0.75	F(2,134) = 0.92, *P* = 0.40
PMC_Thal_AD	1.12E‐03 (4.62E‐05)	1.13E‐03 (4.94E‐05)	1.13E‐03 (4.86E‐05)	43	0.97	0.91	F(2,134) = 0.81, *P* = 0.45^b^
PMC_Thal_FA	4.92E‐01 (2.84E‐02)	4.94E‐01 (2.90E‐02)	4.90E‐01 (2.89E‐02)	43	0.92	0.79	F(2,134) = 3.04, *P* = 0.05
PMC_Thal_MD	7.04E‐04 (2.31E‐05)	7.05E‐04 (2.41E‐05)	7.08E‐04 (2.14E‐05)	43	0.91	0.78	F(2,134) = 2.08, *P* = 0.13^b^
PMC_Thal_RD	4.96E‐04 (2.48E‐05)	4.95E‐04 (2.48E‐05)	4.99E‐04 (2.21E‐05)	43	0.87	0.68	F(2,134) = 2.83, *P* = 0.06
S1_Thal_AD	1.14E‐03 (4.70E‐05)	1.15E‐03 (4.83E‐05)	1.15E‐03 (4.40E‐05)	43	0.97	0.90	F(2,134) = 3.16, *P* = 0.05
S1_Thal_FA	4.78E‐01 (2.65E‐02)	4.78E‐01 (2.38E‐02)	4.79E‐01 (2.42E‐02)	43	0.91	0.76	F(2,134) = 0.03, *P* = 0.97
S1_Thal_MD	7.27E‐04 (2.53E‐05)	7.29E‐04 (2.65E‐05)	7.29E‐04 (2.34E‐05)	43	0.94	0.83	F(2,134) = 0.31, *P* = 0.73
S1_Thal_RD	5.20E‐04 (2.53E‐05)	5.20E‐04 (2.52E‐05)	5.19E‐04 (2.38E‐05)	43	0.9	0.75	F(2,134) = 0.08, *P* = 0.92

Note that all variables are shown as the mean for each timepoint across participants.

AD = Axial Diffusivity; CST = Corticospinal Tract; FA = Fractional Anisotropy; ICC = Intra‐class coefficient; M1 = Primary Motor Cortex; MD = Mean Diffusivity; PMC=Premotor Cortex; RD = Radial Diffusivity.; S1 = Primary Somatosensory Cortex; SD = Standard Deviation.

a Denotes a difference that remains statistically significant following a Bonferroni correction for multiple comparisons, c = 16.

b Cases where the main‐effect of time is superseded by a significant scanner by time interaction; these statistically significant interactions are presented in Appendix S1.

**TABLE 3:** Reliability of Cortical Thickness Measures

Cortical thickness measures	Time1	Time2	Time3	Missing cases	ICC	ICC	Main effect of time
	mean (SD)	mean (SD)	mean (SD)		(2,k)	(2,1)	
Left Hemisphere BA1	2.35 (0.18)	2.36 (0.18)	2.34 (0.19)	21	0.95	0.86	F(2,178) = 1.61, *P* = 0.20
Right Hemisphere BA1	2.41 (0.19)	2.41 (0.18)	2.39 (0.18)	21	0.95	0.85	F(2,178) = 2.93, *P* = 0.06
Left Hemisphere BA2	2.25 (0.13)	2.26 (0.14)	2.24 (0.13)	21	0.94	0.83	F(2,178) = 1.27, *P* = 0.28
Right Hemisphere BA2	2.15 (0.16)	2.15 (0.16)	2.13 (0.16)	21	0.93	0.83	F(2,178) = 2.33, *P* = 0.10
Left Hemisphere BA3a	1.69 (0.13)	1.69 (0.14)	1.68 (0.14)	21	0.98	0.93	F(1.83,163.11) = 2.12, *P* = 0.12
Right Hemisphere BA3a	1.68 (0.20)	1.68 (0.20)	1.67 (0.19)	21	0.99	0.96	F(2,178) = 0.44, *P* = 0.65
Left Hemisphere BA3b	1.89 (0.13)	1.89 (0.12)	1.88 (0.14)	21	0.93	0.93	F(2,178) = 0.46, *P* = 0.63
Right Hemisphere BA3b	1.77 (0.22)	1.76 (0.22)	1.77 (0.21)	21	0.98	0.94	F(2,178) = 1.34, *P* = 0.27
Left Hemisphere BA4a	2.69 (0.17)	2.69 (0.17)	2.67 (0.19)	21	0.88	0.72	F(1.86,165.15) = 1.66, *P* = 0.20
Right Hemisphere BA4a	2.66 (0.21)	2.66 (0.21)	2.65 (0.21)	21	0.9	0.75	F(1.82,162.00) = 1.05, *P* = 0.35
Left Hemisphere BA4p	2.47 (0.20)	2.47 (0.21)	2.49 (0.20)	21	0.86	0.66	F(1.85,164.64) = 1.01, *P* = 0.37
Right Hemisphere BA4p	2.38 (0.20)	2.39 (0.20)	2.38 (0.20)	21	0.82	0.6	F(2,178) = 0.10, *P* = 0.91
Left Hemisphere BA6	2.71 (0.15)	2.71 (0.14)	2.69 (0.16)	21	0.95	0.87	F(2,178) = 3.85, *P* = 0.02
Right Hemisphere BA6	2.69 (0.14)	2.70 (0.14)	2.68 (0.14)	21	0.95	0.87	F(2,178) = 4.11, *P* = 0.02

Note that all variables are shown as the mean for each timepoint across participants.

BA = Brodmann Area; BA1, 2, 3a, 3b = Somatosensory Cortex; BA4a, 4p = Primary Motor Cortex; BA6 = Premotor Cortex.; ICC = Intra‐class coefficient; SD = Standard Deviation.

**TABLE 4:** Reliability of Cortical Volume Measures

Cortical volume measures	Time1	Time2	Time3	Missing cases	ICC	ICC	Main effect of time
	mean (SD)	mean (SD)	mean (SD)		(2,k)	(2,1)	
Left Hemisphere BA1	1860 (264)	1857 (271)	1805 (288)	23	0.97	0.92	F(1.70,147.61) = 18.82, *P* < 0.001^a^ ^,^ ^b^
Right Hemisphere BA1	1640 (288)	1634 (281)	1596 (277)	24	0.98	0.94	F(1.69,145.23) = 19.34, *P* < 0.001^a^ ^,^ ^b^
Left Hemisphere BA2	5944 (1026)	5947 (1031)	5888 (1022)	23	0.99	0.98	F(2,174) = 4.11, *P* = 0.02
Right Hemisphere BA2	4631 (842)	4634 (871)	4580 (865)	23	0.99	0.97	F(2,174) = 3.84, *P* = 0.02
Left Hemisphere BA3a	873 (134)	877 (137)	884 (139)	23	0.99	0.96	F(2,174) = 3.67, *P* = 0.03
Right Hemisphere BA3a	928 (206)	930 (198)	938 (202)	23	0.99	0.98	F(2,174) = 3.44, *P* = 0.03
Left Hemisphere BA3b	3211 (436)	3208 (426)	3165 (441)	23	0.98	0.95	F(2,174) = 6.69, *P* = 0.002
Right Hemisphere BA3b	2543 (448)	2522 (441)	2514 (451)	23	0.99	0.96	F(2,174) = 3.10, *P* = 0.05
Left Hemisphere BA4a	3106 (398)	3101 (409)	3038 (414)	23	0.97	0.92	F(1.70,148.02) = 11.43, *P* < 0.001^a^
Right Hemisphere BA4a	2889 (429)	2883 (424)	2824 (412)	23	0.98	0.93	F(1.75,151.8) = 10.80, *P* < 0.001^a^
Left Hemisphere BA4p	2034 (276)	2041 (301)	2047 (270)	23	0.97	0.9	F(2,174) = 0.44, *P* = 0.65^b^
Right Hemisphere BA4p	1900 (319)	1901 (316)	1894 (305)	23	0.97	0.9	F(2,174) = 0.10, *P* = 0.90
Left Hemisphere BA6	19066 (2433)	19045 (2490)	18674 (2564)	23	0.98	0.95	F(2,174) = 21.51, *P* < 0.001^a^ ^,^ ^b^
Right Hemisphere BA6	16014 (2174)	15985 (2189)	1599 (2187)	23	0.99	0.96	F(1.71,148.55) = 19.83, *P* < 0.001^a^ ^,^ ^b^

Note that all variables are shown as the mean for each timepoint across participants.

BA = Brodmann Area; BA1, 2, 3a, 3b = Somatosensory Cortex; BA4a, 4p = Primary Motor Cortex; BA6 = Premotor Cortex.; ICC = Intra‐class coefficient; SD = Standard Deviation.

a Denotes a difference that remains statistically significant following a Bonferroni correction for multiple comparisons, c = 14.

b Cases where the main‐effect of time is superseded by a significant scanner by time interaction; these statistically significant interactions are presented in Appendix S1.

As shown in Tables 2, 3, 4, there were also statistically significant main‐effects of time for several of the neuroimaging measures. For structural connectivity measures (Table 2), very few outcomes showed statistically significant main‐effects of time. Indeed, following a Bonferroni correction for multiple comparisons, the only statistically significant main‐effect of time was for axial diffusivity between M1 and the thalamus (*P* < 0.001). However, this interaction was superseded by a significant Scanner × Time interaction (*P* < 0.001). Scanner × Time interactions were also found for axial diffusivity and mean diffusivity between PMC and thalamus (*P* < 0.001). (Details of these interactions are shown in Appendix S1.) For cortical thickness measures (Table 3), there were no statistically significant effects of time after adjusting for multiple comparisons. Further, there were no statistically significant Scanner × Time interactions (see Appendix S1).For cortical volume measures (Table 4), following correction for multiple comparisons, there were statistically significant main‐effects of time for BA1 (left and right hemispheres, *P*'s < 0.001), BA4a (left and right hemispheres, *P*'s < 0.001), and BA6 (left and right hemispheres, *P*'s < 0.001). There were also statistically significant Scanner × Time interactions for BA4 and BA6 (left and right hemispheres, *P*'s < 0.001; see Appendix S1).

### Assessing Agreement Between Study Sites

The majority of diffusivity measures (Table 5) differed significantly between scanners/sites (Leiden and Vancouver vs. London and Paris). Specifically, diffusion values were higher for data collected on Siemens scanners compared to those from Philips scanners for AD and MD measures in the M1 tract (*P* < 0.001), AD and MD measures in the PMC tract (*P* < 0.001), and for AD and MD measures in the S1 tract (*P* < 0.001).

**TABLE 5:** Effects of Scanner and Site on Connectivity

	Scanner A (Philips)			Lei v. V	Scanner B (Siemens)			Lo v. *P*	Main effect of scanner		
Variable	Overall	Leiden (Lei)	Vancouver (V)	*P*‐value	Overall	London (Lo)	Paris (P)	*P*‐value	F‐value	df	*P*‐value
CST_AD	1.1E‐03 (3.4E‐5)	1.2E‐03 (3.3E‐5)	1.1E‐03 (3.5E‐5)	0.068	1.2E‐03 (3.3E‐5)	1.2E‐03 (2.6E‐5)	1.2E‐03 (3.2E‐5)	<0.001^a^	20.17	1,62	<0.001^a^
CST_FA	5.2E‐01 (2.1E‐2)	5.2E‐01 (1.8E‐2)	5.2E‐01 (2.4E‐2)	0.806	5.3E‐01 (2.0E‐2)	5.3E‐01 (1.9E‐2)	5.3E‐01 (2.1E‐2)	0.165	5.76	1,62	0.019
CST_MD	7.0E‐04 (2.1E‐5)	7.1E‐04 (2.1E‐5)	6.9E‐04 (1.9E‐5)	0.053	7.2E‐04 (1.6E‐5)	7.1E‐04 (1.3E‐5)	7.3E‐04 (1.6E‐5)	0.006	9.86	1,62	0.003
CST_RD	4.8E‐04 (2.2E‐5)	4.8E‐04 (2.1E‐5)	4.7E‐04 (2.2E‐5)	0.236	4.8E‐04 (1.8E‐5)	4.8E‐04 (1.7E‐5)	4.8E‐04 (1.9E‐5)	0.431	0.46	1,62	0.499
M1_Thal_AD	1.1E‐03 (3.5E‐5)	1.1E‐03 (3.7E‐5)	1.1E‐03 (3.2E‐5)	0.526	1.2E‐03 (3.9E‐5)	1.1E‐03 (2.9E‐5)	1.2E‐03 (3.6E‐5)	<0.001^a^	39.93	1,67	<0.001^a^
M1_Thal_FA	4.7E‐01 (2.4E‐2)	4.66E‐01 (2.5E‐2)	4.7E‐01 (2.2E‐2)	0.647	4.8E‐01 (2.4E‐2)	4.7E‐01 (2.0E‐2)	4.9E‐01 (2.6E‐2)	0.019	4.98	1,67	0.029
M1_Thal_MD	7.0E‐04 (2.0E‐5)	7.0E‐04 (2.0E‐5)	6.9E‐04 (1.9E‐5)	0.145	7.3E‐04 (1.8E‐5)	7.2E‐04 (1.5E‐5)	7.4E‐04 (1.6E‐5)	<0.001^a^	31.05	1,67	<0.001^a^
M1_Thal_RD	5.1E‐04 (2.4E‐5)	5.1E‐04 (2.4E‐5)	5.0E‐04 (2.2E‐5)	0.163	5.2E‐04 (2.0E‐5)	5.2E‐04 (1.8E‐5)	5.2E‐04 (2.1E‐5)	0.658	4.51	1,67	0.037
PMC_Thal_AD	1.1E‐03 (3.6E‐5)	1.1E‐03 (3.6E‐5)	1.1E‐03 (3.6E‐5)	0.556	1.1E‐03 (3.8E‐5)	1.1E‐03 (2.9E‐5)	1.2E‐03 (4.1E‐5)	<0.001^a^	33.02	1,67	<0.001^a^
PMC_Thal_FA	4.8E‐01 (2.5E‐2)	4.8E‐01 (2.5E‐2)	4.8E‐01 (2.5E‐2)	0.531	5.0E‐01 (2.5E‐2)	4.9E‐01 (2.4E‐2)	5.1E‐01 (2.7E‐2)	0.050	9.69	1,67	0.003
PMC_Thal_MD	6.9E‐04 (1.9E‐5)	7.0E‐04 (1.8E‐5)	6.9E‐04 (1.8E‐5)	0.080	7.1E‐04 (1.7E‐5)	7.1E‐04 (1.7E‐5)	7.2E‐04 (1.5E‐5)	0.020	19.86	1,67	<0.001^a^
PMC_Thal_RD	4.9E‐04 (2.2E‐5)	5.0E‐04 (2.2E‐5)	4.9E‐04 (2.0E‐5)	0.086	5.0E‐04 (2.1E‐5)	5.0E‐04 (2.2E‐5)	5.0E‐04 (2.0E‐5)	0.965	0.45	1,67	0.507
S1_Thal_AD	1.1E‐03 (4.0E‐5)	1.1E‐03 (4.3E‐5)	1.1E‐03 (3.4E‐5)	0.198	1.2E‐03 (3.4E‐5)	1.1E‐03 (2.5E‐5)	1.2E‐03 (3.1E‐5)	<0.001^a^	30.87	1,67	<0.001^a^
S1_Thal_FA	4.7E‐01 (2.3E‐2)	4.7E‐01 (2.2E‐2)	4.7E‐01 (2.5E‐2)	0.770	4.8E‐01 (2.2E‐2)	4.7E‐01 (1.7E‐2)	4.9E‐01 (2.4E‐2)	0.008	2.21	1,67	0.142
S1_Thal_MD	7.1E‐04 (2.2E‐5)	7.2E‐04 (2.3E‐5)	7.1E‐04 (2.0E‐5)	0.153	7.4E‐04 (1.6E‐5)	7.3E‐04 (1.5E‐5)	7.4E‐04 (1.5E‐5)	0.020	21.71	1,67	<0.001^a^
S1_Thal_RD	5.1E‐04 (2.4E‐5)	5.2E‐04 (2.2E‐5)	5.1E‐04 (2.4E‐5)	0.339	5.2E‐04 (1.9E‐5)	5.2E‐04 (1.8E‐5)	5.2E‐04 (2.1E‐5)	0.879	3.26	1,67	0.075

Note that all variables are shown as the mean (standard deviation) across three timepoints.

Df = degrees of freedom.

a Denotes a difference that remains statistically significant following a Bonferroni correction for multiple comparisons, c = 16. Tests of site differences within a scanner (Leiden = Lei; London = Lo; Paris = P; Vancouver = V) were based on Fisher's LSD, collapsing across the three different timepoints.

Cortical thickness (Table 6) also differed significantly between scanners bilaterally across most cortical regions (*P*'s < 0.001). The only exceptions were BA4a and BA4p in the left (*P* = 0.064, *P* = 0.046) and the right hemispheres (*P* = 0.132, *P* = 0.631). For all other cortical thickness measurements, values were higher bilaterally for BA1, BA2, BA3a, BA3b, and BA6 for Siemens compared to Philips scanners.

**TABLE 6:** Effects of Scanner and Site on Cortical Thickness

	Scanner A (Philips)			Lei v. V	Scanner B (Siemens)			Lo v. *P*	Main effect of scanner		
Variable	Overall	Leiden (Lei)	Vancouver (V)	*P*‐value	Overall	London (Lo)	Paris (P)	*P*‐value	F‐value	df	*P*‐value
*Left Hemisphere*											
lh_BA1	2.25 (0.14)	2.25 (0.13)	2.26 (0.16)	0.752	2.44 (0.15)	2.48 (0.14)	2.41 (0.15)	0.073	38.63	1,89	<0.001^a^
lh_BA2	2.18 (0.12)	2.20 (0.11)	2.15 (0.12)	0.138	2.31 (0.10)	2.31 (0.07)	2.31 (0.12)	0.939	29.68	1,89	<0.001^a^
lh_BA3a	1.62 (0.13)	1.64 (0.11)	1.58 (0.15)	0.120	1.75 (0.13)	1.75 (0.10)	1.75 (0.15)	0.900	28.74	1,89	<0.001^a^
lh_BA3b	1.81 (0.10)	1.80 (0.08)	1.83 (0.11)	0.388	1.95 (0.12)	1.94 (0.12)	1.95 (0.12)	0.701	39.77	1,89	<0.001^a^
lh_BA4a	2.65 (0.18)	2.66 (0.16)	2.64 (0.21)	0.635	2.71 (0.15)	2.73 (0.14)	2.70 (0.16)	0.477	3.53	1,89	0.064
lh_BA4p	2.44 (0.20)	2.46 (0.18)	2.39 (0.22)	0.213	2.51 (0.18)	2.54 (0.17)	2.48 (0.17)	0.269	4.10	1,89	0.046
lh_BA6	2.61 (0.11)	2.62 (0.11)	2.61 (0.11)	0.874	2.78 (0.12)	2.79 (0.09)	2.78 (0.15)	0.826	46.51	1,89	<0.001^a^
*Right Hemisphere*											
rh_BA1	2.33 (0.17)	2.33 (0.15)	2.33 (0.19)	0.959	2.46 (0.16)	2.50 (0.13)	2.43 (0.18)	0.135	14.57	1,89	<0.001^a^
rh_BA2	2.10 (0.13)	2.10 (0.14)	2.10 (0.13)	0.972	2.19 (0.15)	2.19 (0.12)	2.19 (0.17)	0.933	9.10	1,89	0.003
rh_BA3a	1.61 (0.11)	1.61 (0.12)	1.61 (0.10)	0.970	1.73 (0.21)	1.69 (0.10)	1.78 (0.28)	0.097	10.29	1,89	0.002^a^
rh_BA3b	1.67 (0.14)	1.67 (0.16)	1.67 (0.11)	0.960	1.85 (0.21)	1.82 (0.13)	1.89 (0.26)	0.223	22.00	1,89	<0.001^a^
rh_BA4a	2.63 (0.17)	2.63 (0.17)	2.62 (0.18)	0.795	2.69 (0.20)	2.70 (0.21)	2.67 (0.19)	0.054	2.31	1,89	0.132
rh_BA4p	2.38 (0.18)	2.37 (0.17)	2.39 (0.19)	0.765	2.39 (0.17)	2.39 (0.17)	2.40 (0.18)	0.874	0.23	1,89	0.631
rh_BA6	2.62 (0.11)	2.62 (0.12)	2.62 (0.11)	0.839	2.75 (0.12)	2.77 (0.09)	2.74 (0.14)	0.358	28.7	1,89	<0.001^a^

Note that all variables are shown as the mean (standard deviation) across three timepoints.

Df = degrees of freedom.

a Denotes a difference that remains statistically significant following a Bonferroni correction for multiple comparisons, c = 14. Tests of site differences within a scanner (Leiden = Lei; London = Lo; Paris = P; Vancouver = V) weere based on Fisher's LSD, collapsing across the three different timepoints.

**FIGURE 1:**
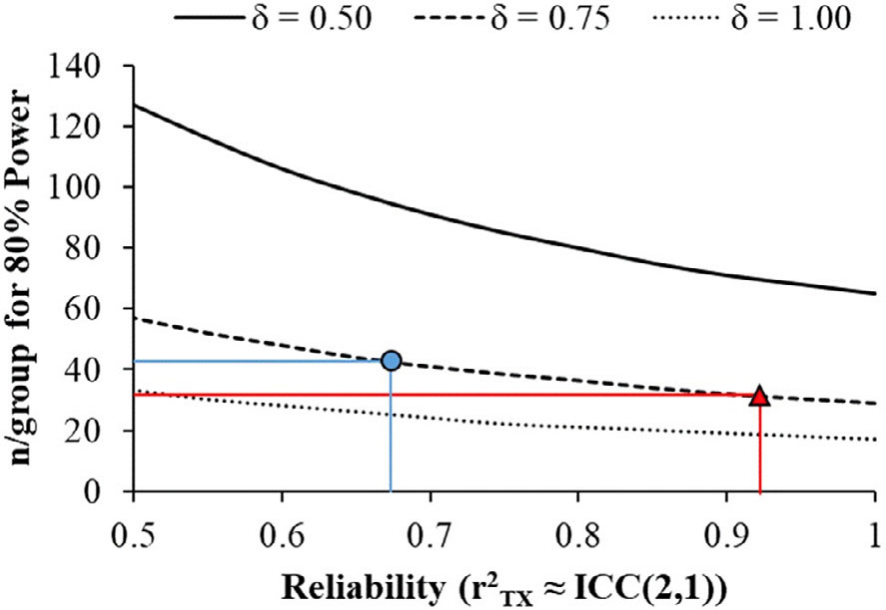
Power analysis: The number of participants per group required to achieve 80% statistical power as a function of a hypothetical underlying effect‐size (δ) and the reliability of the measurement. Reliability is expressed as the ratio of true score variance (T) to observed score variance (X), $r_{\mathrm{TX}}^{2}=\mathrm{var}\left(T\right)/\mathrm{var}\left(X\right)$, which is approximated by the ICC (2,1).

For cortical volume measurements (Table 7), values were higher for Siemens scanners for bilateral BA3a, BA3b, and BA6 (*P*'s < 0.001). Left hemisphere BA1 showed a significant main‐effect of Scanner (*P* < 0.001), whereas right hemisphere BA1 did not (*P* = 0.023). In these regions, volume measures on Siemens scanners were generally higher than on Philips scanners (*P* < 0.05?). Other regions showed a similar pattern, but the differences were not statistically significant following a correction for multiple comparisons.

**TABLE 7:** Effects of Scanner and Site on Cortical Volumes

	Scanner A (Philips)			Lei v. V	Scanner B (Siemens)			Lo v. *P*	Main effect of scanner		
Variable	Overall	Leiden (Lei)	Vancouver (V)	*P*‐value	Overall	London (Lo)	Paris (P)	*P*‐value	F‐value	df	*P*‐value
*Left Hemisphere*											
lh_BA1	1.8E+3 (2.2E+2)	1.7E+3 (2.0E+2)	1.8E+3 (2.4E+2)	0.321	2.0E+3 (3.0E+2)	2.0E+3 (3.0E+2)	2.0E+3 (3.0E+2)	0.094	23.16	1,87	<0.001^a^
lh_BA2	5.8E+3 (8.4E+2)	5.7E+3 (7.8E+2)	5.9E+3 (9.0E+2)	0.503	6.3E+3 (1.1E+3)	6.0E+3 (1.0E+3)	6.7E+3 (1.1E+3)	<0.001^a^	5.93	1,87	0.017
lh_BA3a	8.4E+2 (1.2E+2)	8.4E+2 (1.2E+2)	8.4E+2 (1.2E+2)	0.316	9.4E+2 (1.2E+2)	9.4E+2 (1.3E+2)	9.4E+2 (1.0E+2)	0.679	20.46	1,87	<0.001^a^
lh_BA3b	3.1E+3 (3.6E+2)	3.1E+3 (3.7E+2)	3.1E+3 (3.6E+2)	0.973	3.4E+3 (5.1E+2)	3.4E+3 (5.7E+2)	3.4E+3 (4.4E+2)	0.051	17.63	1,87	<0.001^a^
lh_BA4a	3.0E+3 (4.2E+2)	3.0E+3 (4.2E+2)	3.0E+3 (4.1E+2)	0.807	3.2E+3 (3.5E+2)	3.2E+3 (3.5E+2)	3.2E+3 (3.6E+2)	0.747	6.64	1,87	0.012
lh_BA4p	2.1E+3 (3.2E+2)	2.1E+3 (2.9E+2)	2.0E+3 (3.5E+2)	0.010	2.1E+3 (2.2E+2)	2.1E+3 (2.6E+2)	2.1E+3 (1.7E+2)	0.560	2.49	1,87	0.118
lh_BA6	1.8E+4 (2.0E+3)	1.8E+4 (2.0E+3)	1.9E+4 (2.2E+3)	0.285	2.0E+4 (2.5E+3)	2.0E+4 (2.4E+3)	2.0E+4 (2.6E+3)	0.399	22.68	1,87	<0.001^a^
*Right Hemisphere*											
rh_BA1	1.6E+3 (2.5E+2)	1.5E+3 (2.7E+2)	1.6E+3 (2.2E+2)	0.776	1.7E+3 (3.1E+2)	1.7E+3 (3.4E+02)	1.7E+3 (2.8E+2)	0.778	5.37	1,86	0.023
rh_BA2	4.7E+3 (8.3E+2)	4.5E+3 (8.8E+2)	4.9E+3 (7.4E+2)	0.195	4.7E+3 (9.2E+2)	4.6E+3 (9.1E+2)	4.8E+3 (9.3E+2)	0.242	0.01	1,87	0.953
rh_BA3a	8.8E+2 (1.2E+2)	8.6E+2 (1.1E+2)	9.0E+2 (1.2E+2)	0.51	1.0E+3 (2.2E+2)	9.8E+2 (1.9E+2)	1.0E+3 (2.5E+2)	0.303	13.55	1,87	<0.001^a^
rh_BA3b	2.4E+3 (3.4E+2)	2.4E+3 (3.8E+2)	2.5E+3 (3.0E+2)	0.842	2.7E+3 (4.7E+2)	2.7E+3 (4.9E+2)	2.8E+3 (4.4E+2)	0.398	14.29	1,87	<0.001^a^
rh_BA4a	2.8E+3 (3.4E+2)	2.8E+3 (4.0E+2)	2.8E+3 (2.8E+2)	0.998	3.0E+3 (4.3E+2)	3.0E+3 (4.2E+2)	3.0E+3 (4.6E+2)	0.304	8.33	1,87	0.005
rh_BA4p	1.9E+3 (2.5E+2)	1.9E+3 (2.7E+2)	1.9E+3 (2.2E+2)	0.803	2.0E+3 (3.2E+2)	1.9E+3 (2.3E+2)	2.0E+3 (3.9E+2)	0.661	3.38	1,87	0.069
rh_BA6	1.5E+4 (1.9E+3)	1.5E+4 (1.8E+3)	1.6E+4 (2.0E+3)	0.189	1.7E+4 (2.3E+3)	1.7E+4 (2.0E+3)	1.7E+4 (2.6E+3)	0.155	14.56	1,87	<0.001^a^

Note that all variables are shown as the mean (standard deviation) across three timepoints.

Df = degrees of freedom.

a Denotes a difference that remains statistically significant following a Bonferroni correction for multiple comparisons, c = 14. Tests of site differences within a scanner (Leiden = Lei; London = Lo; Paris = P; Vancouver = V) were based on Fisher's LSD, collapsing across the three different timepoints.

Within the connectivity measures, there were no statistically significant differences between the Leiden and Vancouver (Philips scanner) sites; however, there were significant differences between London and Paris (Siemens scanner) sites for AD and/or MD measures of all tracts (*P*'s < 0.001). For the cortical thickness measures, there were no statistically significant differences between sites with the same scanner type (*P* ≥ 0.05). For cortical volume measures, the only statistically significant differences were in left BA2, in which the Paris site had significantly higher average volume than the London site (*P* < 0.001).

## Discussion

MRI measures of brain morphology and anatomical connectivity are key to the characterization of biological mechanisms associated with neurodegenerative disease. Understanding the reliability of these MRI measures can help interpret any pathology‐related changes, and also inform the power required for their use in a study or trial. In this study we investigated the reliability of morphology measures: cortical thickness and volume; and anatomical connectivity measured using DTI within the sensorimotor network over time, in a group of healthy individuals from the multisite, multiscanner Track‐On HD study. All measures of cortical thickness, cortical volume, and white matter diffusivity for both hemispheres showed high levels of reliability, suggesting that differences between measurements over time represent real systematic changes rather than inherent methodological variability. However, while there was consistency between sites using the same scanner, there were clear systematic differences for all structural measures according to scanner type, thus highlighting the importance of this issue when planning multisite studies using different MRI scanners.

We first examined the long‐term stability of morphological MRI‐derived measures of cortical thickness and volume for brain regions within the sensorimotor cortex. Reproducibility for almost all regions was high. This was true for both reliability across the three timepoints (ICC(2,k)) and the estimated variation in “true” values captured by a single timepoint (ICC (2,1)). This is consistent with previous studies that have tested the reliability of Freesurfer‐derived cortical thickness measures in healthy people, with reproducibility across a number of visits ranging from just 2 to up to 10.^26^, ^27^ Similarly, when examining anatomical connectivity using diffusivity metrics extracted from white matter sensorimotor pathways, we found generally high levels of reliability across three timepoints (ICC(2,k)), but some diffusivity measures were only moderately reliable at any given individual timepoint (ICC (2,1)). Both ICC measures showed that axial diffusivity was most reliable across tracts, with radial diffusivity generally lowest, but improved by calculating an average across several measurements. Measures of diffusion‐based anatomical connectivity are less likely to be used in clinical trials, but they provide very useful indications of network breakdown due to changes in white matter microstructure. Previous studies have shown good levels of reliability, but tended to focus on the robustness of measures within regions of interest rather than tractography‐based analyses.^11^, ^12^, ^13^ Furthermore, previous studies have generally tested reliability over a short time period (eg, hours or days). While studies of reliability over a short timescale are important, reliability is an emergent property of the measurement tool and the context in which it is used,^14^ so these studies have reduced generalizability to large multisite studies with considerable time between scans (eg, weeks or months). Our study, therefore, has, albeit retrospectively, examined variability in structural measures with greater generalizability to long timescales, specifically, studies with annual timepoints.

Understanding the magnitude of variation from different sources is useful for researchers planning multisite and/or longitudinal studies. Reliability of a given measurement has important implications for statistical power, and the number of participants needed to achieve a desired level of statistical power is a function of the level of significance, the desired power, and the underlying effect size. However, this effect size assumes no measurement error (unless based on empirical data) and very few constructs are measured that precisely.^28^ In many cases, researchers estimate effect‐sizes with heuristics (eg, Cohen's d = 0.5 is a “moderate” effect; Cohen's d = 1.0 is a “large” effect), which makes the implicit assumption of no measurement error. As such, it is important that researchers temper their predicted effect‐sizes by incorporating measurement error.^29^, ^30^ For example, assuming alpha = 0.05 and a population difference of δ = 0.75 between groups, most of the connectivity/thickness/volume measures in the present study would have ≥80% statistical power to detect a difference between groups when *n*/group ≅ 30 (See Figure 1). If the primary outcome measure in a study were *M1‐Thalamus AD,* then the observed ICC (2,1) was 0.92 participants, per group would be required to achieve 80% statistical power because the error in this measure effectively reduces the effect‐size from an idealized δ = 0.75 to d = 0.72. The ICC (2,1) reflects the average ratio of true variance to total variance captured by any single measurement. That is, if the ratio of the variance in “true” scores (T) to observed scores (X) is r^2^_tx_ = var(T)/var(X) = 0.25 in the population, ICC (2,1) will approximate 0.25 (large samples) regardless of the number of observations made. As such, ICC (2,1) reflects the average amount of variance in true scores that is captured by a single measurement. Presenting ICC (2,1) as a complement to ICC(2,k) is important because ICC(2,k) is sensitive to the number of measurements, whereas ICC (2,1) is not and, unless experimenters are making a fixed number of repeated assessments, ICC (2,1) is most relevant to trial designs with a single pre‐ and posttest assessment.

Alternatively, if the primary outcome measure was *PMC‐Thalamus RD,* then the observed ICC (2,1) was 0.68. This would mean that 43 participants are required per group, because the measurement error reduces the idealized δ = 0.75 to d = 0.62. Thus, the *n*/group required to achieve statistical power varies markedly within these connectivity measures, because the reliability of a measure at a given timepoint (ICC (2,1)) varied so substantially. Naturally, as the effect‐size increases (eg, from δ = 0.5 to 1.0) the *n*/group required to achieve 80% power decreases. However, these data show that despite generally good reliability across these neuroimaging measures, the differences in reliability still have negative consequences for statistical power depending on the outcome.

We also examined the amount of change in our measures over 2 years. Cortical volume measures tended to show reliable decreases over time, consistent with previous research for healthy adults in this age range.^31^, ^32^ For example, BA1 *volume* decreased by 3%, and BA4a/BA6 *volume* decreased by 2% on average. While these effect‐sizes are relatively small, the high reliability (ie, low within‐subject variance) provided adequate statistical power to detect change. Cortical thickness, however, did not show statistically significant changes over time, suggesting that the magnitude of change may be smaller than that of cortical volume. It is also likely that Freesurfer measurements of cortical thickness are less reliable, given that small errors in segmentation can significantly inflate thickness values for particular regions where volumetric measures are more robust to this type of error. This could potentially impact detection of the subtle changes in thickness that may occur over a 2‐year period in healthy controls.

Our data were collected on two different types of MRI scanners and despite good within‐participant reliability, there was an effect of scanner type for most measures. There appeared to be a consistent trend for sites using Siemens scanners to produce larger values for connectivity, cortical thickness, and cortical volume measures than sites using Philips scanners. For volumetric and cortical thickness measures, differences between Philips and Siemens scanners are unsurprising, as FreeSurfer was developed primarily on Siemens and GE scanners with the application on Philips data tested later in development.^33^ It is also important to note that this study was not designed to test differences between scanners or study sites, so these differences must be interpreted with caution. Different participants were measured at the different study sites, so these between‐site differences are not measures of “interrater” reliability. That said, we believe that the between‐scanner differences do reflect real differences between scanners and not merely sampling variability, given that there is demographic and anthropomorphic similarity between participants across study sites and that within‐scanner differences were not statistically different (which would be more suggestive of differences due to sampling variability alone). Potvin et al showed that scanner type is responsible for up to 2.8% of the variance (right caudate), with most regions showing variability as low as 0.1%.^34^, ^35^ This is a relatively small proportion of the variance, particularly when compared with age, sex, and total intracranial volume. In the current study, we have shown a clear effect of scanner type and, qualitatively, our results tend to agree with those of Duchesne et al, with higher volumes being reported for Siemens scanners compared with Philips scanners.^36^ It is difficult to compare quantitatively our results, given that Potvin et al's study scanned one individual, where we have larger samples, but not multiple scans per person on different scanners. Taken together, however, these results further suggest our scanner differences are real and not due to different samples at different sites.

These potential differences are especially relevant when comparing results between studies or planning multisite trials, as researchers need to account for between‐site differences when pooling data sources (eg, using multilevel modeling procedures^37^) or when contrasting data from different sources.

Finally, we investigated differences between sites using the same scanner. Between‐site differences were generally less pronounced than between‐scanner differences. For example, there were no differences between the two sites using Philips scanners, although there were still some notable differences in diffusivity measures between sites using Siemens scanners. Again, we must be cautious when interpreting these findings, given that the design was not balanced (ie, sites were nested within scanner types and ideally we would have all participants at each site scanned with each scanner, fully crossing the effects of site and scanner). In addition, these were relatively underpowered tests compared to those of between‐scanner differences. It is clear, therefore, that despite phantom scanning, rigorous multiscanner quality control and standardization of training, there were still significant differences between scanners (for many measures) and between sites for a particular scanner (mostly for diffusivity measures). This reinforces the importance of thorough training and streamlining of scanning protocols and using analyses that can account for between‐site differences in large multicenter studies and clinical trials using MRI scanning as endpoint measures.

It is important to note that these between‐site (or between‐scanner) comparisons are not measures of “interrater” reliability, because different participants were measured at the different study sites. However, these measures are still informative because it is important to understand systematic between‐participant and within‐participant variability in our data.

Quantifying sources of between‐ and within‐subject variability in older adults over a long timescale has important implications for clinical neuroscience. Large observational studies, for instance in Huntington's disease or Friedreich's ataxia, suggest that longitudinal studies with a long timescale (eg, over a number of years) are important in understanding disease‐related brain alterations.^4^, ^38^ Differences in data collection techniques, equipment, and procedures could introduce variation/noise over real biological changes that occur over time. For our healthy cohort, structural measures of thickness and volume were generally robust over time, although impacted by scanner type and, therefore, also potentially suitable for use in a clinical trial as an exploratory endpoint. Diffusion metrics do not have the same level of robustness, as they are seemingly more affected by scanner type and in terms of intersite variability. Therefore, when embarking on a longitudinal study, it is crucial to have some knowledge of the potential variability that may be introduced when investigating a brain structure or connectivity.

## Track‐On HD Investigators

A. Coleman, J. Decolongon, M. Fan, T. Koren (University of British Columbia, Vancouver); C. Jauffret, D. Justo, S. Lehericy, K. Nigaud, R. Valabrègue (ICM and APHP, Pitié‐ Salpêtrière University Hospital, Paris); S. Klöppel, E. Scheller, L. Minkova (University of Freiberg, Freiberg), A. Schoonderbeek, E. P 't Hart (Leiden University Medical Centre, Leiden); C. Berna, M. Desikan, R. Ghosh, D. Hensman Moss, E. Johnson, P. McColgan, G. Owen, M. Papoutsi, A. Razi, J. Read, (University College London, London); D. Craufurd (Manchester University, Manchester); R. Reilmann, N. Weber (George Huntington Institute, Munster); H. Johnson, J.D. Long, J. Mills (University of Iowa, Iowa); J. Stout, I. Labuschagne (Monash University, Melbourne); G.B. Landwehrmeyer, I. Mayer (Ulm University, Ulm).

## Funding

This work was funded by the CHDI Foundation and the Wellcome Trust (G.R.). S.J.T. is partly supported by the UK Dementia Research Institute that receives its funding from DRI Ltd., funded by the UK Medical Research Council, Alzheimer's Society, and Alzheimer's Research UK. Some of this work was also undertaken at UCLH/UCL, who acknowledge support from the Department of Health's NIHR Biomedical Research Centre.

K.L. is supported by the Canadian Institutes of Health Research (PTJ 153330) and the Auburn University Internal Grants Program (170138). S.G., R.S., G.R., and S.T. receive support from a Wellcome Trust Collaborative Award (200181/Z/15/Z). All authors declare that they have no conflicts of interest.

## Supporting information

**Appendix S1.** Supplementary Material.
