# Supplementary material for: Longitudinal Structural MRI in Neurologically Healthy Adults
Source: J Magn Reson Imaging. 2020 May 29;52(5):1385–99. doi: 10.1002/jmri.27203 (PMC8425332; doi:10.1002/jmri.27203)
Supplement: Supplementary file 1 — Appendix S1. Supplementary Material. [file JMRI-52-1385-s001.docx]

**Supplementary Table 1.** Descriptive statistics for the diffusivity measures showing variation between- and within-participants over time.

| **Measure** | **Overall Mean** | **Between SD** | **Average Within SD** | **Average Within CoV** |
| --- | --- | --- | --- | --- |
| CST_AD | 1.18E-03 | 3.87E-05 | 1.21E-05 | 1.03E-02 |
| CST_FA | 5.26E-01 | 2.13E-02 | 9.41E-03 | 1.80E-02 |
| CST_MD | 7.13E-04 | 2.05E-05 | 8.64E-06 | 1.21E-02 |
| CST_RD | 4.80E-04 | 2.06E-05 | 1.02E-05 | 2.12E-02 |
| M1_Thal_AD | 1.12E-03 | 4.86E-05 | 1.27E-05 | 1.13E-02 |
| M1_Thal_FA | 4.76E-01 | 2.45E-02 | 1.03E-03 | 2.18E-02 |
| M1_Thal_MD | 7.17E-04 | 2.42E-05 | 8.82E-06 | 1.23E-02 |
| M1_Thal_RD | 5.14E-04 | 2.23E-02 | 1.06E-05 | 2.06E-02 |
| PMC_Thal_AD | 1.13E-03 | 4.67E-05 | 1.29E-05 | 1.14E-02 |
| PMC_Thal_FA | 4.92E-01 | 2.68E-02 | 1.14E-02 | 2.33E-02 |
| PMC_Thal_MD | 7.06E-04 | 2.11E-05 | 9.58E-06 | 1.36E-02 |
| PMC_Thal_RD | 4.96E-04 | 2.13E-05 | 1.17E-05 | 2.35E-02 |
| S1_Thal_AD | 1.15E-03 | 4.50E-05 | 1.28E-05 | 1.12E-02 |
| S1_Thal_FA | 4.78E-01 | 2.28E-02 | 1.05E-02 | 2.20E-02 |
| S1_Thal_MD | 7.28E-04 | 2.36E-05 | 8.90E-06 | 1.22E-02 |
| S1_Thal_RD | 5.20E-04 | 2.26E-05 | 1.05E-05 | 2.20E-02 |

Note all values have been rounded to the nearest hundredth. Overall mean is calculated as the mean of $x_{ij}$ , where i indexes the participant number and j indexes the time-point. Between SD is the between-participants standard deviation (i.e., the standard deviation of $\bar{x}_{i}$). Average within SD is the average of the within-participant standard deviations (i.e., the average of the standard deviation of $x_{j}$ for each participant). Average within CoV is the average within-participant coefficient of variation, which is calculated by dividing the within-participant standard deviation by the mean for that participant and then taking the average of those individual CoVs.

**Supplementary Table 2.** Descriptive statistics for cortical thickness measures showing variation between- and within-participants over time.

| **Measure** | **Overall Mean** | **Between SD** | **Average Within SD** | **Average Within CoV** |
| --- | --- | --- | --- | --- |
| Left BA1 Thickness | 2.353 | 0.174 | 0.054 | 0.023 |
| Right BA1 Thickness | 2.400 | 0.174 | 0.057 | 0.024 |
| Left BA2 Thickness | 2.250 | 0.126 | 0.046 | 0.021 |
| Right BA2 Thickness | 2.146 | 0.148 | 0.040 | 0.025 |
| Left BA3a Thickness | 1.688 | 0.135 | 0.033 | 0.020 |
| Right BA3a Thickness | 1.677 | 0.192 | 0.034 | 0.020 |
| Left BA3b Thickness | 1.886 | 0.122 | 0.045 | 0.024 |
| Right BA3b Thickness | 1.768 | 0.210 | 0.046 | 0.026 |
| Left BA4a Thickness | 2.683 | 0.158 | 0.074 | 0.028 |
| Right BA4a Thickness | 2.657 | 0.189 | 0.079 | 0.030 |
| Left BA4p Thickness | 2.475 | 0.479 | 0.092 | 0.038 |
| Right BA4p Thickness | 2.384 | 0.171 | 0.095 | 0.041 |
| Left BA6 Thickness | 2.704 | 0.143 | 0.045 | 0.017 |
| Right BA6 Thickness | 2.690 | 0.135 | 0.042 | 0.016 |

Note all values have been rounded to the nearest hundredth. Overall mean is calculated as the mean of $x_{ij}$ , where i indexes the participant number and j indexes the time-point. Between SD is the between-participants standard deviation (i.e., the standard deviation of $\bar{x}_{i}$). Average within SD is the average of the within-participant standard deviations (i.e., the average of the standard deviation of $x_{j}$ for each participant). Average within CoV is the average within-participant coefficient of variation, which is calculated by dividing the within-participant standard deviation by the mean for that participant and then taking the average of those individual CoVs.

**Supplementary Table 3.** Descriptive statistics for cortical volume measures showing variation between- and within-participants over time.

| **Measure** | **Overall Mean** | **Between SD** | **Average Within SD** | **Average Within CoV** |
| --- | --- | --- | --- | --- |
| Left BA1 Volume | 1840.86 | 268.25 | 63.12 | 0.04 |
| Right BA1 Volume | 1627.53 | 273.94 | 56.22 | 0.04 |
| Left BA2 Volume | 5926.07 | 1017.63 | 132.56 | 0.02 |
| Right BA2 Volume | 4631.38 | 842.03 | 120.77 | 0.03 |
| Left BA3a Volume | 878.00 | 134.65 | 23.55 | 0.03 |
| Right BA3a Volume | 932.48 | 201.30 | 26.34 | 0.03 |
| Left BA3b Volume | 3194.60 | 426.80 | 83.92 | 0.03 |
| Right BA3b Volume | 2524.40 | 443.75 | 70.36 | 0.03 |
| Left BA4a Volume | 3081.42 | 396.87 | 97.86 | 0.03 |
| Right BA4a Volume | 2874.92 | 404.52 | 89.31 | 0.03 |
| Left BA4p Volume | 2040.82 | 273.20 | 69.38 | 0.03 |
| Right BA4p Volume | 1904.36 | 299.43 | 73.35 | 0.04 |
| Left BA6 Volume | 18928.40 | 2462.98 | 447.60 | 0.02 |
| Right BA6 Volume | 15928.98 | 2152.19 | 359.05 | 0.02 |

Note all values have been rounded to the nearest hundredth. Overall mean is calculated as the mean of $x_{ij}$ , where i indexes the participant number and j indexes the time-point. Between SD is the between-participants standard deviation (i.e., the standard deviation of $\bar{x}_{i}$). Average within SD is the average of the within-participant standard deviations (i.e., the average of the standard deviation of $x_{j}$ for each participant). Average within CoV is the average within-participant coefficient of variation, which is calculated by dividing the within-participant standard deviation by the mean for that participant and then taking the average of those individual CoVs.

**Supplementary Table 4.** Intraclass correlation coefficients for MRI measures across different study sites. Note all values have been rounded to the nearest hundredth.

|  | **ICC(2,1)** |  |  |  | **ICC(2,k)** |  |  |  |
| --- | --- | --- | --- | --- | --- | --- | --- | --- |
| **MRI Measure** | **Leiden** | **London** | **Paris** | **Vancouver** | **Leiden** | **London** | **Paris** | **Vancouver** |
| ***Diffusivity Measures*** |  |  |  |  |  |  |  |  |
| CST_AD | 0.71 | 0.85 | 0.88 | 0.59 | 0.88 | 0.95 | 0.96 | 0.81 |
| CST_FA | 0.54 | 0.77 | 0.75 | 0.89 | 0.78 | 0.91 | 0.90 | 0.96 |
| CST_MD | 0.72 | 0.65 | 0.77 | 0.82 | 0.88 | 0.85 | 0.91 | 0.93 |
| CST_RD | 0.66 | 0.68 | 0.66 | 0.91 | 0.85 | 0.86 | 0.86 | 0.97 |
| M1_Thal_AD | 0.87 | 0.87 | 0.81 | 0.70 | 0.95 | 0.95 | 0.93 | 0.87 |
| M1_Thal_FA | 0.67 | 0.83 | 0.76 | 0.47 | 0.86 | 0.94 | 0.91 | 0.85 |
| M1_Thal_MD | 0.74 | 0.74 | 0.79 | 0.80 | 0.90 | 0.90 | 0.92 | 0.92 |
| M1_Thal_RD | 0.65 | 0.75 | 0.75 | 0.84 | 0.85 | 0.90 | 0.90 | 0.94 |
| PMC_Thal_AD | 0.84 | 0.80 | 0.91 | 0.81 | 0.94 | 0.92 | 0.97 | 0.93 |
| PMC_Thal_FA | 0.60 | 0.79 | 0.85 | 0.76 | 0.82 | 0.92 | 0.94 | 0.83 |
| PMC_Thal_MD | 0.66 | 0.66 | 0.75 | 0.82 | 0.85 | 0.85 | 0.90 | 0.93 |
| PMC_Thal_RD | 0.53 | 0.70 | 0.75 | 0.80 | 0.77 | 0.87 | 0.90 | 0.92 |
| S1_Thal_AD | 0.91 | 0.82 | 0.80 | 0.36 | 0.97 | 0.93 | 0.92 | 0.63 |
| S1_Thal_FA | 0.62 | 0.67 | 0.79 | 0.87 | 0.83 | 0.86 | 0.92 | 0.95 |
| S1_Thal_MD | 0.85 | 0.73 | 0.71 | 0.73 | 0.94 | 0.89 | 0.88 | 0.89 |
| S1_Thal_RD | 0.74 | 0.69 | 0.73 | 0.85 | 0.89 | 0.87 | 0.89 | 0.94 |
|  |  |  |  |  |  |  |  |  |
| ***Cortical Thickness Measures*** | **Leiden** | **London** | **Paris** | **Vancouver** | **Leiden** | **London** | **Paris** | **Vancouver** |
| lh BA1 | 0.70 | 0.78 | 0.86 | 0.89 | 0.87 | 0.91 | 0.95 | 0.96 |
| rh BA1 | 0.83 | 0.64 | 0.88 | 0.91 | 0.93 | 0.85 | 0.96 | 0.97 |
| lh BA2 | 0.76 | 0.66 | 0.84 | 0.83 | 0.91 | 0.85 | 0.94 | 0.93 |
| rh BA2 | 0.79 | 0.74 | 0.86 | 0.85 | 0.92 | 0.90 | 0.95 | 0.95 |
| lh BA3a | 0.88 | 0.84 | 0.93 | 0.95 | 0.96 | 0.94 | 0.98 | 0.98 |
| rh BA3a | 0.93 | 0.88 | 0.98 | 0.90 | 0.97 | 0.96 | 0.99 | 0.97 |
| lh BA3b | 0.71 | 0.76 | 0.70 | 0.91 | 0.88 | 0.90 | 0.88 | 0.97 |
| rh BA3b | 0.94 | 0.84 | 0.94 | 0.88 | 0.98 | 0.94 | 0.98 | 0.96 |
| lh BA4a | 0.62 | 0.80 | 0.62 | 0.84 | 0.83 | 0.92 | 0.83 | 0.94 |
| rh BA4a | 0.61 | 0.55 | 0.41 | 0.59 | 0.82 | 0.79 | 0.67 | 0.81 |
| lh BA4p | 0.72 | 0.74 | 0.36 | 0.80 | 0.89 | 0.90 | 0.63 | 0.93 |
| rhBA4p | 0.57 | 0.61 | 0.55 | 0.75 | 0.80 | 0.83 | 0.78 | 0.90 |
| lh BA6 | 0.70 | 0.79 | 0.91 | 0.83 | 0.87 | 0.92 | 0.97 | 0.93 |
| rh BA6 | 0.77 | 0.82 | 0.91 | 0.79 | 0.91 | 0.93 | 0.97 | 0.92 |
|  |  |  |  |  |  |  |  |  |
| ***Cortical Volume Measures*** | **Leiden** | **London** | **Paris** | **Vancouver** | **Leiden** | **London** | **Paris** | **Vancouver** |
| lh BA1 | 0.76 | 0.93 | 0.97 | 0.90 | 0.91 | 0.98 | 0.99 | 0.97 |
| rh BA1 | 0.89 | 0.97 | 0.97 | 0.87 | 0.96 | 0.99 | 0.99 | 0.95 |
| lh BA2 | 0.94 | 0.98 | 0.98 | 0.97 | 0.98 | 0.99 | 0.99 | 0.99 |
| rh BA2 | 0.96 | 0.98 | 0.97 | 0.95 | 0.99 | 0.99 | 0.99 | 0.98 |
| lh BA3a | 0.96 | 0.96 | 0.95 | 0.89 | 0.97 | 0.99 | 0.98 | 0.96 |
| rh BA3a | 0.95 | 0.98 | 0.99 | 0.81 | 0.98 | 0.99 | 0.99 | 0.93 |
| lh BA3b | 0.92 | 0.96 | 0.92 | 0.95 | 0.97 | 0.99 | 0.97 | 0.98 |
| rh BA3b | 0.96 | 0.98 | 0.94 | 0.94 | 0.99 | 0.99 | 0.98 | 0.98 |
| lh BA4a | 0.88 | 0.95 | 0.90 | 0.95 | 0.96 | 0.98 | 0.97 | 0.98 |
| rh BA4a | 0.87 | 0.95 | 0.95 | 0.91 | 0.95 | 0.98 | 0.98 | 0.97 |
| lh BA4p | 0.94 | 0.91 | 0.69 | 0.95 | 0.98 | 0.97 | 0.87 | 0.98 |
| rhBA4p | 0.89 | 0.87 | 0.91 | 0.92 | 0.96 | 0.95 | 0.97 | 0.97 |
| lh BA6 | 0.88 | 0.98 | 0.97 | 0.92 | 0.96 | 0.99 | 0.99 | 0.97 |
| rh BA6 | 0.88 | 0.98 | 0.99 | 0.93 | 0.96 | 0.99 | 0.99 | 0.98 |
|  |  |  |  |  |  |  |  |  |
| ***Overall – Median [IQR]*** | 0.78  [0.69, 0.89] | 0.81  [0.74, 0.94] | 0.86  [0.75, 0.94] | 0.86  [0.80, 0.91] | 0.92  [0.87, 0.96] | 0.93  [0.90, 0.98] | 0.95  [0.90, 0.98] | 0.95  [0.93, 0.97] |

Supplementary Table 5. Scanner by Time Interactions in the Repeated Measures ANOVAs for structural neuroimaging measures.

| **MRI Measure** | **Time by Scanner Interaction** | **Post-Hoc Effect of Time** | |
| --- | --- | --- | --- |
| ***Diffusivity Measures*** |  | Phillips | Siemens |
| CST_AD | F(2,124) = 0.94, p = 0.391 |  |  |
| CST_FA | F(2,124) = 3.14, p = 0.047 | p = 0.042 | p = 0.617 |
| CST_MD | F(2,124) = 1.86, p = 0.160 |  |  |
| CST_RD | F(2,124) = 2.51, p = 0.085 |  |  |
| M1_Thal_AD | F(2,134) = 11.68, p < 0.001* | p = 0.770 | p < 0.001 |
| M1_Thal_FA | F(2,134) = 1.92, p = 0.150 |  |  |
| M1_Thal_MD | F(2,134) = 5.21, p = 0.007 | p = 0.288 | p < 0.001 |
| M1_Thal_RD | F(2,134) = 1.63, p = 0.200 |  |  |
| PMC_Thal_AD | F(2,134) = 7.03, p = 0.001* | p = 0.383 | p < 0.001 |
| PMC_Thal_FA | F(2,134) = 2.45, p = 0.090 |  |  |
| PMC_Thal_MD | F(2,134) = 7.35, p = 0.001* | p = 0.041 | p = 0.003 |
| PMC_Thal_RD | F(2,134) = 4.06, p = 0.019 | p = 0.039 | p = 0.210 |
| S1_Thal_AD | F(2,134) = 2.02, p = 0.137 |  |  |
| S1_Thal_FA | F(2,134) = 0.46, p = 0.631 |  |  |
| S1_Thal_MD | F(2,134) = 1.84, p = 0.162 |  |  |
| S1_Thal_RD | F(2,134) = 0.83, p = 0.445 |  |  |
|  |  |  |  |
| ***Cortical Thickness Measures*** |  |  |  |
| lh BA1 | F(2,178) = 0.03, p = 0.969 |  |  |
| rh BA1 | F(2,178) = 0.73, p = 0.484 |  |  |
| lh BA2 | F(2,178) = 0.28, p = 0.757 |  |  |
| rh BA2 | F(2,178) = 0.25, p = 0.778 |  |  |
| lh BA3a | F(2,178) = 4.64, p = 0.011 | p = 0.001 | p = 0.725 |
| rh BA3a | F(2,178) = 1.07, p = 0.345 |  |  |
| lh BA3b | F(2,178) = 0.12, p = 0.890 |  |  |
| rh BA3b | F(2,178) = 0.29, p = 0.747 |  |  |
| lh BA4a | F(2,178) = 0.13, p = 0.882 |  |  |
| rh BA4a | F(2,178) = 0.28, p = 0.757 |  |  |
| lh BA4p | F(2,178) = 0.01, p = 0.992 |  |  |
| rhBA4p | F(2,178) = 1.32, p = 0.270 |  |  |
| lh BA6 | F(2,178) = 3.13, p = 0.047 | p = 0.010 | p = 0.681 |
| rh BA6 | F(2,178) = 2.02, p = 0.135 |  |  |
|  |  |  |  |
| ***Cortical Volume Measures*** |  |  |  |
| lh BA1 | F(2,174) = 8.02, p < 0.001* | p < 0.001 | p = 0.121 |
| rh BA1 | F(2,172) = 9.30, p < 0.001* | p < 0.001 | p = 0.254 |
| lh BA2 | F(2,174) = 1.75, p = 0.178 |  |  |
| rh BA2 | F(2,174) = 0.50, p = 0.608 |  |  |
| lh BA3a | F(2,174) = 0.41, p = 0.667 |  |  |
| rh BA3a | F(2,174) = 5.41, p = 0.005 | p < 0.001 | p = 0.769 |
| lh BA3b | F(2,174) = 3.85, p = 0.023 | p < 0.001 | p = 0.846 |
| rh BA3b | F(2,174) = 2.59, p = 0.078 |  |  |
| lh BA4a | F(2,174) = 4.97, p = 0.008 | p < 0.001 | p = 0.360 |
| rh BA4a | F(2,174) = 3.76, p = 0.025 | p < 0.001 | p < 0.001 |
| lh BA4p | F(2,174) = 0.04, p = 0.963 |  |  |
| rhBA4p | F(2,174) = 1.56, p = 0.212 |  |  |
| lh BA6 | F(2,174) = 12.21, p < 0.001* | p < 0.001 | p = 0.210 |
| rh BA6 | F(2,174) = 10.87, p < 0.001* | p < 0.001 | p = 0.157 |

* Denotes an interaction that remains statistically significant following a Bonferroni correction for multiple comparisons. Post-hoc tests are presented for both cases for completeness, but to be conservative we only interpret the post-hoc tests for interactions following correction for multiple comparisons.
